# Acute Kidney Injury Due to Nephrolithiasis in Autosomal Dominant Polycystic Kidney Disease Treated With Tolvaptan: A Case Report

**DOI:** 10.7759/cureus.62597

**Published:** 2024-06-18

**Authors:** Hisato Shima, Masaaki Nishitani, Hirofumi Izaki, Jun Minakuchi

**Affiliations:** 1 Kidney Disease, Kawashima Hospital, Tokushima, JPN; 2 Nephrology and Hypertension, Kamei Hospital, Tokushima, JPN; 3 Urology, Kawashima Hospital, Tokushima, JPN; 4 Urology, Tokushima Prefectural Central Hospital, Tokushima, JPN

**Keywords:** tolvaptan, nephrolithiasis, transurethral lithotripsy, acute kidney injury, autosomal dominant polycystic kidney disease

## Abstract

A 61-year-old asymptomatic female with autosomal dominant polycystic kidney disease (ADPKD) on tolvaptan therapy was hospitalized for acute kidney injury (AKI). Nephrolithiasis had already been diagnosed; however, the patient had not undergone any interventions. She also presented with hyponatremia possibly caused by overhydration. Because the estimated glomerular filtration rate (eGFR) decline was significantly higher than the predicted rate, we considered a possible case of postrenal AKI and examined computed tomography (CT), which revealed left hydronephrosis with a 9.4-mm ureteric stone at the level of L3/L4. We restricted fluid intake, which resulted in an increase in sodium levels. She was treated with transurethral lithotripsy (TUL) twice, which successfully improved her kidney function. Although the serum sodium levels increase because of aquaresis in almost all patients treated with tolvaptan, our case was unique in that the patient presented with hyponatremia. We should pay more attention to the periodical follow-up of nephrolithiasis in addition to the increase in total kidney volume and decide the appropriate time to treat nephrolithiasis depending on the case. We should also keep in mind that ADPKD patients have a high frequency of nephrolithiasis and, even if asymptomatic, investigate urinary tract obstruction and hydronephrosis in case of AKI.

## Introduction

Autosomal dominant polycystic kidney disease (ADPKD) is the most common hereditary kidney disease, accounting for 5%-10% of end-stage renal diseases [1]. Due to urinary stasis and metabolic and anatomical abnormalities, the prevalence of nephrolithiasis in ADPKD is 8%-36%, which is 5-10 times higher than that in the general population [2,3]. Flank pain due to nephrolithiasis is commonly reported in patients with ADPKD [4]. However, nearly half of the cases with nephrolithiasis in ADPKD patients are asymptomatic [2]. Most of the stones in ADPKD patients were located in the renal calyces, where they would be less likely to be symptomatic [5]. The prevalence of stone interventions is 1%-8% in patients with ADPKD [5]. The application of transurethral lithotripsy (TUL) for nephrolithiasis and postrenal acute kidney injury (AKI) has rarely been reported in ADPKD patients treated with tolvaptan. Tolvaptan, a selective vasopressin V2 receptor antagonist, increases urinary water excretion [6]. Side effects include urinary frequency, hypernatremia, osmotic demyelination injury, and acute liver injury.

Herein, we report the case of an asymptomatic patient with ADPKD treated with tolvaptan who presented with AKI due to nephrolithiasis. This case highlights the importance of assessing ADPKD patients for AKI and discussing the appropriate time to treat nephrolithiasis.

## Case presentation

**Figure 1:**
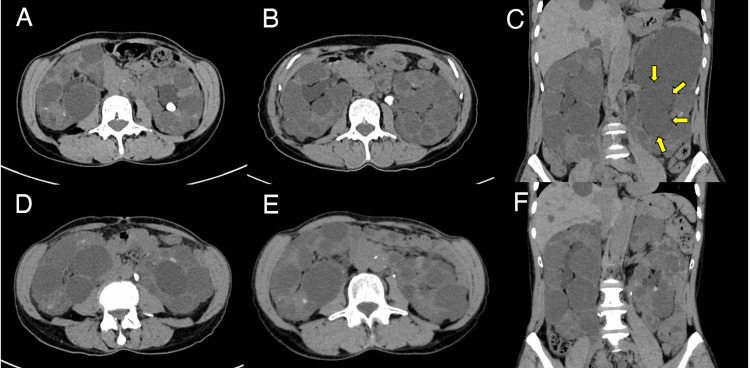
Patient's abdominal CT Abdominal CT revealed (A) a 10.5 mm renal pelvic stone and (B) a 10.3 mm stone in the ureteropelvic junction. CT revealed (C) left hydronephrosis (arrows) and (D) a 9.4 mm ureteric stone at the level of L3/L4. (E and F) A second TUL successfully fragmented and extracted almost all the stone fragments and improved left hydronephrosis. CT: computed tomography, TUL: transurethral lithotripsy

A 61-year-old female was hospitalized for AKI and had a medical history of ADPKD. The left and right kidney volumes were 1,079.8 mL and 760.4 mL, respectively. She began tolvaptan treatment (60 mg/day) with an estimated glomerular filtration rate (eGFR) of 38.5 mL/minute/1.73 m^2 ^16 months before hospitalization. Computed tomography (CT) revealed a non-obstructing 10.5 mm renal pelvic stone 13 months before hospitalization (Figure 1A) and a 10.3 mm stone in the ureteropelvic junction seven months before hospitalization (Figure 1B).

**Figure 2:**
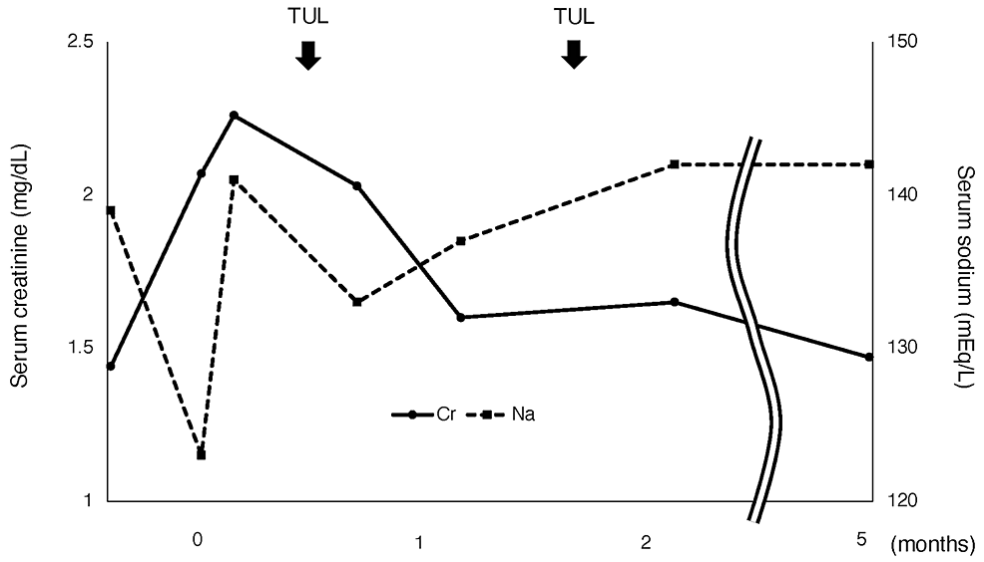
Clinical course of the patient TUL: transurethral lithotripsy, Cr: creatinine, Na: sodium

Since the stone was asymptomatic, we decided that no intervention was required. Her serum creatinine (sCr) and sodium levels were 1.44 mg/dL and 139 mEq/L, respectively, two weeks before hospitalization. On admission, her vital signs were as follows: body temperature of 36.5°C, blood pressure of 128/85 mmHg, pulse of 86/minute, and respiratory rate of 16/minute. She gained weight of about 2 kg and presented pitting peripheral edema. Her daily water intake was 4,000-4,500 mL/day. There was no noticeable steady decrease in her urine output. Blood tests revealed elevated sCr (2.07 mg/dL) and hyponatremia (sodium: 123 mEq/L) (Figure 2).

Urinalysis revealed hematuria (sediment red blood cells: 50-99 per high-power field), pyuria (sediment white blood cells: 10-19 per high-power field), and proteinuria (1.87 g/day). CT revealed left hydronephrosis with a 9.4 mm ureteric stone at the L3/L4 level (Figure 1C, 1D). Retrograde pyelography revealed complete unilateral obstruction. After we restricted her fluid intake for seven days (1,500-1,800 mL/day), her sodium levels gradually increased (Figure 2). Based on the size and location of the stone, we performed TUL with postoperative ureteral stent placement. Because of the residual fragments, we performed a second TUL and successfully fragmented and extracted almost all the stone fragments (Figure 1E). Left hydronephrosis was improved (Figure 1F). Stone analysis revealed calcium oxalate stones. After TUL, there were no complications, and sCr levels improved to 1.47 mg/dL at the five-month follow-up (Figure 2).

## Discussion

Our case report highlights two important clinical recommendations for physicians for the treatment of ADPKD patients. First, physicians should consider the possibility of postrenal AKI, even in asymptomatic ADPKD patients diagnosed with nephrolithiasis in the past. Second, physicians should pay greater attention to the periodic follow-up of nephrolithiasis in addition to the increase in total kidney volume.

AKI is classified as prerenal, renal, or postrenal [7]. This patient presented with hyponatremia, which was very rare in patients with ADPKD during tolvaptan administration. The serum sodium levels increase because of aquaresis in almost all cases treated with tolvaptan. Because we did not examine plasma and urine osmolality, there was a lack of information regarding the differential diagnosis of hyponatremia. Tolvaptan can cause thirst in up to 5% of patients due to water diuresis [8]. Considering the daily water intake and weight gain, a possible cause of hyponatremia in this case was excessive water intake, and prerenal AKI was considered less likely. Therefore, we restricted fluid intake, which resulted in an increase in sodium levels. The Mayo imaging classification is a useful model for predicting disease progression in ADPKD [9]. This case was classified as Mayo class 1C (predicted eGFR decline rate: -2.36 mL/minute/1.73 ^2^/year). On admission, the eGFR decline rate was significantly higher than the predicted rate. It is true that this patient has nephrolithiasis, so it is ordinary to rule out hydronephrosis when eGFR declines rapidly. Therefore, we considered a possible case of renal or postrenal AKI and performed abdominal CT. We should consider that ADPKD patients may have a high frequency of nephrolithiasis, and even if asymptomatic, they should be investigated for urinary tract obstruction and hydronephrosis in case of AKI. Obstruction can also occur due to the cyst compressing the collecting duct and outflow tract. In this case, sCr levels and hydronephrosis improved after TUL. Therefore, we considered that nephrolithiasis mainly attributed the AKI.

Tolvaptan demonstrably slows down the increase in total kidney volume in ADPKD [6]. Doctors treating patients who receive tolvaptan for ADPKD tend to focus more on total kidney volume than on nephrolithiasis when they regularly examine the CT during follow-up for renal volume measurement. Renal volume > 500 mL is a significant risk factor for nephrolithiasis in patients with ADPKD [10]. The predominant size and number of cysts are also considered factors that affect calculi formation [11]. In this case, the left renal volume was 1,079.8 mL, which is a risk factor for nephrolithiasis. Nephrolithiasis is associated with the onset of renal failure in patients with ADPKD [12]. Therefore, physicians should pay greater attention to nephrolithiasis and periodically follow up on the size and location of stones, if present. In ADPKD patients with large stones or symptomatic urolithiasis, the size and location of stones influence the choice of treatment. Currently, three non-invasive or minimally invasive methods are known: extracorporeal shock wave lithotripsy (ESWL), percutaneous nephrolithotomy (PCNL), and TUL [13]. ESWL is the primary management option for smaller upper urinary stones (<20 mm) [14]. However, because of the potential risks of traumatic hemorrhage into the cysts and interference with the passage of stone fragments, the efficacy of ESWL in ADPKD remains controversial [15]. PCNL is recommended for the treatment of large stones (>20 mm) in the upper ureter and kidneys [13]. However, PCNL in ADPKD can be challenging because the calyceal spaces are often narrow and long owing to the compressive effects of cysts [15]. TUL is the treatment of choice for nephrolithiasis (<20 mm) and is relatively safe for patients with ADPKD [15]. Therefore, we treated the patient with TUL.

There is no consensus on the appropriate time for treating non-obstructing asymptomatic nephrolithiasis. The European Association of Urology recommends observation for asymptomatic nephrolithiasis but recommends active stone removal in case of stone growth, stones > 15 mm in size, obstruction, infection, and patient preference [16]. Prophylactic ESWL for small asymptomatic nephrolithiasis (stones < 15 mm) has few advantages [17]. On the contrary, stones > 10 mm are less likely to pass spontaneously and require intervention [18]. Patients with identified risk factors, including stones > 5 mm, diabetes mellitus, hyperuricemia, or non-lower calyceal stones, were more likely to experience stone growth and require treatment [19]. In this case, for stones of 10-11 mm, the patient might have benefited from earlier intervention. However, because of the relative rarity of ADPKD, further studies are needed to investigate which treatments are suitable and when.

## Conclusions

On examining the CT for renal volume measurement in patients with ADPKD, greater attention should be paid to the periodical follow-up for nephrolithiasis. Considering the presented case, it is imperative that doctors consider postrenal AKI in asymptomatic patients with ADPKD treated with tolvaptan. Physicians should also decide the appropriate time for treatment depending on the case.
